# Repeat Emergency Visits for Mental Health Patients: Before and during the Covid19 pandemic

**DOI:** 10.1192/j.eurpsy.2022.707

**Published:** 2022-09-01

**Authors:** R. Tempier, E.M. Bouattane

**Affiliations:** 1 University of Ottawa, Psychiatry, Ottawa, Canada; 2 Montfort Hospital, Administration, ottawa, Canada

## Abstract

**Introduction:**

Frequent users of Emergency Departments (EDs) are a diverse group accounting for disproportionate EDs visits. Psychiatric patients are more likely to visit EDs (Slankamenac, 2020). EDs utilisation by psychiatric patients increased by 4.4% during COVID-19 pandemic.

**Objectives:**

to determine frequent users characteristics within an Ottawa University Hospital, and assess Covid19 impact on overutilization of EDs compared to other hospitals.

**Methods:**

Retrospective study of repeat visits characteristics, data extracted from EMR database. Repeat visits defined as no less than 30 days first visit to any EDs. Period of observation: March 1st, 2018 - February 28th, 2021 Results.

**Results:**

64% EDS visits for MH, 35% for addictions. More men (57%), age groups: 16-34 y.o. (41%), 34-64 y.o. (51%), 65 +y.o. (8%).

Top presenting reasons: suicidality, self-harm, depression (40.5%). Anxiety, situational crisis (16%), bizarre behavior (12%).

Most prevalent diagnoses: schizophrenia (28.7%), stress and anxiety (25.2%), personality disorders (13.5%) and depressive episode (10.6%). Only 35.1% admitted after repeat ED visits, 35.1% came by ambulance. Increase during peak pandemic exceeding 20%. Clearly pandemic created more pressures for MH services needs.

**Conclusions:**

Schizophrenia and personality disorders made most prevalent diagnostic groups. Even when patients are in acute needs, they do not always require hospitalization. Investigating what MH conditions that got more stressed by the Covid19 pandemic will be of interest.

**Disclosure:**

No significant relationships.

